# Human Subtilisin Kexin Isozyme-1 (SKI-1)/Site-1 Protease (S1P) regulates cytoplasmic lipid droplet abundance: A potential target for indirect-acting anti-dengue virus agents

**DOI:** 10.1371/journal.pone.0174483

**Published:** 2017-03-24

**Authors:** Anastasia Hyrina, Fanrui Meng, Steven J. McArthur, Sharlene Eivemark, Ivan R. Nabi, François Jean

**Affiliations:** 1 Department of Microbiology and Immunology, Life Sciences Institute, University of British Columbia, Vancouver, British Columbia, Canada; 2 Department of Cell and Physiological Sciences, Life Sciences Institute, University of British Columbia, Vancouver, British Columbia, Canada; CEA, FRANCE

## Abstract

Viral hijacking and manipulation of host-cell biosynthetic pathways by human enveloped viruses are shared molecular events essential for the viral lifecycle. For *Flaviviridae* members such as hepatitis C virus and dengue virus (DENV), one of the key subsets of cellular pathways that undergo manipulation is the lipid metabolic pathways, underlining the importance of cellular lipids and, in particular, lipid droplets (LDs) in viral infection. Here, we hypothesize that targeting cellular enzymes that act as key regulators of lipid homeostasis and LD formation could represent a powerful approach to developing a novel class of broad-spectrum antivirals against infection associated with all DENV serotypes (1–4) circulating around the world. Using PF-429242, an active-site-directed inhibitor of SKI-1/S1P, we demonstrate that inhibition of SKI-1/S1P enzymatic activity in human hepatoma Huh-7.5.1 cells results in a robust reduction of the LD numbers and LD-positive areas and provides a means of effectively inhibiting infection by DENV (1–4). Pre-treatment of Huh-7.5.1 cells with PF-429242 results in a dose-dependent inhibition of DENV infection [median inhibitory dose (EC_50_) = 1.2 microM; median cytotoxic dose (CC_50_) = 81 microM; selectivity index (SI) = 68)] and a ~3-log decrease in DENV-2 titer with 20 microM of PF-429242. Post-treatment of DENV-2 infected Huh-7.5.1 cells with PF-429242 does not affect viral RNA abundance, but it does compromise the assembly and/or release of infectious virus particles. PF-429242 antiviral activity is reversed by exogenous oleic acid, which acts as an inducer of LD formation in PF-429242-treated and non-treated control cells. Collectively, our results demonstrate that human SKI-1/S1P is a potential target for indirect-acting pan-serotypic anti-DENV agents and reveal new therapeutic opportunities associated with the use of lipid-modulating drugs for controlling DENV infection.

## Introduction

Dengue virus (DENV) represents a significant threat to global public health, with approximately 390 million cases annually and about 2.5 billion people living in endemic countries [1–3]. DENV is the causative agent of dengue fever (DF) and of life-threatening severe dengue, including dengue haemorrhagic fever (DHF) and dengue shock syndrome (DSS) [4]. Although DENV was first isolated more than 70 years ago, current treatment and prevention approaches are still limited to palliative relief of symptoms and vector control [4–7]. Currently, four DENV serotypes (DENV-1 to -4) transmitted by *Aedes aegypti* and *Aedes albopictus* mosquitoes are known to circulate in humans [3, 8]. All four DENV serotypes are considered to be *hyper-endemic* in most tropical and subtropical areas of the world, and they are poised to spread into new territories [3, 9]. A better understanding of host-DENV interactions and DENV pathogenesis is urgently needed to design broad-spectrum antivirals that will be effective against all four DENV serotypes.

The DENV serotypes are members of the *Flavivirus* genus with single-stranded positive-sense RNA genomes encoding three structural proteins (capsid [C], precursor membrane [prM], and envelope [E]) and seven nonstructural proteins (NS1, NS2A, NS2B, NS3, NS4A, NS4B, and NS5) [10]. RNA viruses are associated with intrinsically high rates of mutation, with the DENV-4 evolution rate estimated at 6.89 × 10^−4^ substitutions/site/year [11, 12]. Given the importance of reliably targeting all four DENV serotypes and limiting the formation of antiviral resistance, indirect-acting antivirals (IAA) that interfere with the viral hijacking of host factors important for the viral lifecycle are an attractive therapeutic avenue [13, 14].

Cellular factors such as lipids and cholesterol are involved in every step of the DENV lifecycle [15–19]. Different drugs targeting either lipid or cholesterol pathways have been tested, including an inhibitor of fatty acid synthase (C75), an inhibitor of intra-cellular cholesterol transport (U18666A), inhibitors of cholesterol synthesis (lovastatin, fluvastatin, and pravastatin), and the hypolipidemic agent arachidonic acid 5-lipoxygenase inhibitor (nordihydroguaiaretic acid). All of these inhibitors achieved variable reductions in DENV virus replication or infectious particle formation [20–23], underlining the importance of cellular lipids and, in particular, lipid droplets (LDs) in DENV infection. LDs are dynamic intracellular lipid storage organelles that play multiple roles during the DENV lifecycle [18, 20]. They consist of a neutral lipid core (e.g., triglycerides and cholesterol esters) surrounded by a phospholipid monolayer containing LD-associated proteins such as adipose differentiation-related protein (ADRP) [24].

In this study, we investigated the molecular functions of human subtilisin kexin isozyme-1/site-1 protease (SKI-1/S1P), a key master regulator of the lipid homeostasis/sterol regulatory element-binding protein (SREBP) pathway [25], in the formation of cellular lipid storage droplets and the DENV lifecycle. In mammals, the biosynthesis of cholesterol, fatty acids, and triglycerides is tightly regulated by a family of transcriptional factors called SREBPs. Two genes encode three SREBP isoforms: SREBP-1a, SREBP-1c, and SREBP-2 [26]. SREBP-2 and SREBP-1c are the predominant forms in the liver, regulating genes involved in sterol biosynthesis and fatty acid synthesis, respectively [27]. The inactive precursor of SREBP (pre-SREBP) is synthesized in the endoplasmic reticulum (ER) as a membrane-bound protein. Its activation is dependent on the presence of sterols and requires the cleavage of pre-SREBP to release the mature form, which then translocates to the nucleus. When sterol levels are low, the SREBP cleavage-activating protein (SCAP) escorts SREBP from the ER to the Golgi apparatus, where SREBP is sequentially cleaved by two cellular proteases: SKI-1/S1P and site-2-protease (S2P) [28]. These cleavages liberate the N-terminal fragment of SREBP (n-SREBP), which further translocates into the nucleus to activate genes involved in lipid and cholesterol metabolism, such as low density lipoprotein receptor (LDLR) and proprotein convertase subtilisin/kexin type 9 (PCSK9) [26, 29, 30]. When cellular sterol levels are high, the insulin-induced gene protein (Insig) associates with SCAP, which causes the SCAP–pre-SREBP complex to be retained in the ER, thereby preventing the formation of n-SREBP and decreasing the expression of SREBP target genes [31].

Given that SKI-1/S1P-mediated SREBP proteolytic activation controls the expression of genes directly involved in intracellular fatty acid and cholesterol biosynthesis [28], two important components of LDs, we hypothesized that pharmacological inhibition of the subtilase SKI-1/S1P could represent a powerful approach to developing a novel class of broad-spectrum antivirals against DENV infection. We proposed that strategic manipulation of human SKI-1/S1P enzymatic activity would effectively inhibit DENV infection by blocking cytoplasmic LD formation and interfering with DENV hijacking of cytoplasmic LD, a critical event in the DENV lifecycle [20].

Here using an active-site-directed aminopyrrolidineamide-based inhibitor of SKI-1/S1P, PF-429242 [25], we demonstrated that inhibition of host SKI-1/S1P enzymatic activity effectively blocks DENV (1–4) from establishing infection in human hepatoma Huh-7.5.1 cells. PF-429242 antiviral activity was observed both pre- and post-establishment of viral infection and was associated with a dramatic decrease in LD abundance in PF-429242-treated Huh-7.5.1 cells. Our studies demonstrate SKI-1/S1P’s potential as a novel host-directed pan-serotypic anti-DENV target, and they reveal therapeutic opportunities associated with the use of lipid-modulating drugs for controlling DENV infection.

## Materials and methods

### Cell culture and reagents

Human hepatoma Huh-7.5.1 cells were kindly provided by Dr. Francis Chisari (Scripps Research Institute, La Jolla, CA, USA) [32]. The African green monkey kidney epithelial Vero E6 cell line was obtained from the American Type Culture Collection (ATCC) (ATCC^®^ CC1-81^™^). Cultured cells were maintained in Dulbecco’s Modified Eagle Medium (DMEM) supplemented with 1% penicillin/streptomycin, 1% L-glutamine, 1% nonessential amino acids, 1% 4-(2-hydroxyethyl)-1-piperazineethanesulfonic acid (HEPES), and 10% heat-inactivated fetal bovine serum (FBS) (Gibco/ Invitrogen, Burlington, ON, Canada). Vero E6 cells were cultured in M199 medium (Sigma-Aldrich Corp., St. Louis, MO, USA) supplemented with 1% HEPES, 1% L-glutamine, 1% sodium bicarbonate, and 5% FBS (Gibco). Bovine serum albumin (BSA), oleic acid (OA), and dimethyl sulfoxide (DMSO) were obtained from Sigma-Aldrich Corp.

### Viruses and infections

The four serotypes of DENV were kindly provided by Dr. Mike Drebot from the National Microbiology Laboratory (Winnipeg, MB, Canada): DENV-1, Hawaiian; DENV-2, New Guinea C; DENV-3, H-87; and DENV-4, H-241. Huh-7.5.1 cells were either inoculated with DENV-1, -2, -3, or -4 [multiplicity of infection (MOI) = 1 or 0.01] or mock-infected at 37°C for 1 hour before the inoculum was removed and fresh complete media was added. Cell supernatant, lysates, and/or RNA were collected at various time-points post-infection for analysis by secondary infection/plaque assay, Western blot, and/or reverse transcription quantitative real-time PCR (RT-qPCR), respectively.

### Small-molecule inhibition of SKI-1/S1P

PF-429242 (chemical name: 4-[(Diethylamino)methyl]-*N*-[2-(2-methoxyphenyl)ethyl]-*N*-(3*R*)-3-pyrrolidinylbenzamide), a reversible, competitive small-molecule aminopyrrolidine-amide inhibitor of SKI-1/S1P [13, 25, 33], was synthesized by Dr. Peter Chua at the Center for Drug Research and Development (CDRD) at the University of British Columbia (Vancouver, BC, Canada) according to previously described protocols [34]. The chemical was dissolved in DMSO and stored at a concentration of 100 mM. To serve as a negative control, an intermediate product, acetylated PF-429242 (AcPF-429242), was also synthesized at CDRD. To investigate the antiviral activity of the small molecule, Huh-7.5.1 cells were treated with PF-429242 for 24 hours. Media was then removed and the cells were infected with one of the DENV serotypes (DENV 1–4) for 48 hours. Alternatively, cells were first infected with DENV-2 for 24 hours; then the media was removed and replaced with fresh media supplemented with PF-429242 for 48 hours. DMSO and AcPF-429242-treated cells were used as controls. For lipid supplementation assays [35], Huh-7.5.1 cells were treated with PF-429242 as described above with the addition of 0.6 mM oleic acid (with BSA in molar ratio 6:1) or the equivalent concentration of oleic acid/BSA alone during DENV infection.

### Cytotoxicity assay

Cell viability was determined using CellTiter 96 AQueous One Solution Cell Proliferation Assay (Promega, Madison, WI, USA) following manufacturer’s instructions. Briefly, Huh-7.5.1 cells were treated with different concentrations of PF-429242, AcPF-429242, or corresponding concentrations of DMSO for 24 hours in a 96-well plate. After 24 hours, media containing the inhibitor was removed and fresh media was added to the cells for an additional 48 hours. Following the incubation period, 20 μl of CellTiter 96 Aqueous One Solution Reagent was added to each well containing the samples in 100 μl of culture medium, and the plate was incubated for 1 hour at 37°C. Production of formazan by cells from a tetrazolium compound [3-(4,5-dimethylthiazol-2-yl)-5-(3-carboxymethoxyphenyl)-2-(4-sulfophenyl)-2H-tetrazolium, inner salt; MTS] was detected by measuring absorbance at 490 nm. The 50% cytotoxic concentration (CC_50_) was defined as the compound’s concentration (μM) required to reduce cell viability by 50%, which was calculated by curve fitting from two independent experiments.

### Western blot analysis

Cultured cells were washed twice with ice-cold phosphate buffered saline (PBS) and re-suspended in a cold radioimmunoprecipitation assay (RIPA) buffer [50 mM Tris–HCl (pH 8), 1% Triton X-100, 0.5% sodium deoxycholate, 150 mM NaCl, and 0.1% SDS] containing a 1X complete, EDTA-free, protease inhibitor cocktail (Roche, Laval, QC, Canada). Whole cell extracts were vortexed and then clarified by centrifugation at 12,000×g for 15 minutes. Soluble extracts were mixed with 2X sample buffer (62.5 mM Tris-HCl, pH 6.8, 25% glycerol, 2% SDS, 0.01% bromophenol blue, and 5% β-mercaptoethanol). Samples stained with anti-NS1 antibody were mixed with sample buffer without β-mercaptoethanol. Samples were electrophoresed on 10–15% SDS polyacrylamide gels and transferred to nitrocellulose membranes. Membranes were blocked in Odyssey blocking buffer (LI-COR Biosciences, Lincoln, NE, USA), and proteins of interest were detected by probing with the appropriate primary and secondary antibodies diluted in Odyssey blocking buffer containing 0.1% Tween-20. The membrane was probed using a mouse anti-NS1 (1:50 dilution; Abcam, Cambridge, MA, USA), rabbit anti-β-tubulin (1:3,000 dilution; Abcam), and secondary antibodies IRDye 680-conjugated (red bands) or 800-conjugated (green bands) donkey anti-mouse or goat anti-rabbit antibodies (1:10,000; LI-COR Biosciences). Protein bands were detected and quantified using the Odyssey Infrared Imaging System (LI-COR Biosciences). All immunoblots were scanned at a wavelength of 700 nm for detecting IRDye 680-labeled antibodies and at a wavelength of 800 nm for IRDye 800-conjugated antibodies. Signal intensities were quantified by means of the Odyssey software version 3.0. β-tubulin was consistently used as a loading control and for normalizing protein expression.

### Curve-fitting, half-maximal Effective Concentration (EC_50_) and 50% Cytotoxic Concentration (CC_50_) determination and Selectivity Index (SI)

A custom hyperbolic fit function [y = a+bx/(1+cx)] in Igor Pro software (WaveMetrics, Inc., Portland, OR, USA) was used for fitting DENV-2 NS1 expression and PF-429242 inhibition curves and for determining EC_50_ value. The same function was used for fitting cell viability (O.D. at 490 nm) when treated with PF-429242 or DMSO. The reported EC_50_ and CC_50_ values are the average of the values calculated from at least two independent experiments (± SEM). The selectivity index (SI) was determined as the ratio of CC_50_ to EC_50_ concentration.

### Plaque assay

DENV titers were determined by performing plaque assay as previously described [36]. Briefly, Vero E6 cells monolayers were seeded in 12-well plates (Falcon; Becton Dickinson, Lincoln Park, NJ, USA) and incubated at 37°C in a CO_2_ incubator. Supernatants of DENV or mock-infected Huh-7.5.1 cells were tested using tenfold dilutions starting at 1:10. Plaques were visualized on day 5 by staining with 4% neutral red solution (Sigma-Aldrich Corp.) in PBS. Statistical significance was calculated by a student’s *t*-test (paired) based on three independent experiments.

### RNA isolation

Total RNA was isolated using a miniRNeasy kit (QIAGEN, Mississauga, ON, Canada) according to the manufacturer’s instructions. The concentration and purity of RNA were determined by a NanoDrop ND-1000 Spectrophotometer (Thermo Scientific, Nepean, ON, Canada).

### Quantitative Real-Time PCR (qRT-PCR)

500 ng of purified total RNA was reverse-transcribed to cDNA using TaqMan reverse transcription reagents (random hexamers; Applied Biosystems, Foster City, CA, USA). Quantitative RT-PCR was carried out on an Mx3005P real-time PCR system (Stratagene, La Jolla, CA, USA) using Brilliant III Fast QPCR reagents (Stratagene) according to the manufacturer’s instructions. DENV RNA was analyzed using a previously reported serotype-specific DENV primer probe set [37] (S1 Fig). DENV RNA levels were quantified across samples and normalized to β-actin RNA levels using 500 nM primers (forward: 5′-GCC CTG AGG CAC TCT TCC-3′ and reverse: 5′-GGA TGT CCA CGT CAC ACT TC-3′) and 250 nM probe (5′-AC TCC ATG CCC AGG AAG GAA GGC-3′ with a 5′ Cy5 fluorophore and 3′ black hole quencher). The expression levels of six cellular mRNAs were quantified by qRT-PCR using TaqMan gene expression assays [Applied Biosystems, assays ID (Hs00965485_g1:FURIN; Hs00545399_m1:PCSK9; Hs00921626_m1:SKI-1/S1P; Hs01092524_m1:LDLR; Hs01081784_m1: SREBP-2; Hs01088691_m1:SREBP-1c]. For data analysis, the 2^-ΔΔCt^ method was used, and mean fold changes in expression are shown relative to mock or control treated samples. The data were analyzed with one-way or two-way ANOVAs.

### Confocal microscopy

Huh-7.5.1 cells seeded in μ-Slide 8 Well IbiTreat (Ibidi, Madison, WI, USA) were fixed in 3% v/v paraformaldehyde in PBS, then permeabilized in PBS containing 0.01% digitonin or 0.1% Triton X-100. Cells were probed with primary rabbit anti-capsid (C) antibody [20] (1:1000, kindly provided by Dr. Andrea V. Gamarnik (Fundación Instituto Leloir-CONICET, Argentina)), then incubated with secondary antibodies (Alexa 647 conjugated goat anti-rabbit), Hoechst dye, and Nile red dye diluted in PBS. Nile red, 9-diethylamino-5H-benzo[alpha]phenoxazine-5-one, is a selective fluorescent stain for detecting intracellular lipid droplets [38]. The wells were then imaged using Leica SP8 confocal microscope (Leica Microsystems, Wetzlar, Germany) with a 100x objective (HC PL APO 100x/1.40 OIL). All quantified images were acquired using the same laser intensity and gain settings, and LDs were enumerated by applying the same threshold setting to each image. For the LD analysis based on Nile red fluorescence, the calculated values reported for the LD-positive area (μm^2^) per cell are means ± SEM (>50 cells analyzed) and the numbers of LDs per cell are means ± SEM (>50 cells analyzed).

## Results

### Inhibition of SKI-1/S1P enzymatic activity using PF-429242 impairs activation of the SREBP pathway and correlates with a dramatic decrease in lipid droplet number and area

**Fig 1:**
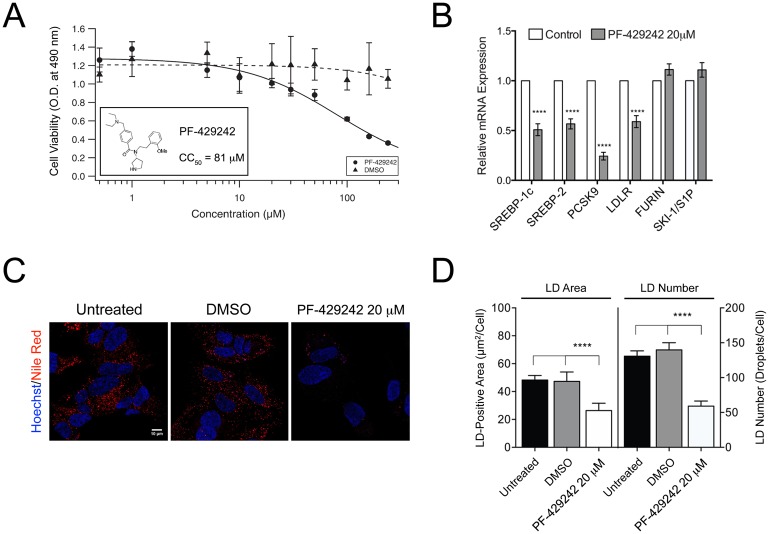
Inhibition of SKI-1/S1P enzymatic activity using PF-429242 impairs activation of the SREBP pathway and correlates with a dramatic decrease in lipid droplet number and area. (A–D) Huh-7.5.1 cells were treated with various concentrations of PF-429242 and corresponding concentrations of DMSO for 24 hours before the inhibitor was removed and fresh complete media was added to the cells for an additional 48 hours. (A) The relative cytotoxicity of the compound was determined using an MTS-based cell viability assay (CC_50_ = 81 μM). The absorbance measured at 490 nm is proportional to the number of living cultured cells. (B) Total RNA was extracted, and the mRNA levels of SREBP-1c, SREBP-2, PCSK9, LDLR, FURIN, and SKI-1/S1P were quantified by qRT-PCR. Results were normalized against β-actin mRNA levels and expressed as fold change. Statistical significance was calculated for PF-429242-treated cells (20 μM) compared to 0.02% DMSO-treated cells (control) with a two-way ANOVA for each mRNA presented. (C) Representative images of the effect of PF-429242 (20 μM) and 0.02% DMSO on lipid droplets are shown. Fixed cells were permeabilized with Triton X-100 and stained for cell nuclei using Hoechst dye and for lipid droplets using Nile red. Images were obtained using a Leica SP8 confocal microscope with a 100x objective. (D) Abundance of LDs was quantified by measuring the average LD-positive area (μm^2^)/cell and the average number of LDs/cell based on Nile red fluorescence in untreated, 0.02% DMSO-treated, and PF-429242-treated cells (20 μM) using Fiji software (>50 cells analyzed). Statistical significance was calculated with a two-way ANOVA with a Bonferroni’s post-test. Values represent average ± SEM of three independent experiments (****, *p* < 0.001).

To test our hypothesis that strategic manipulation of SKI-1/S1P dependent activation of the SREBP pathway would effectively inhibit DENV infection by blocking cytoplasmic LD formation, we first investigated the effect of the small-molecule SKI-1/S1P inhibitor PF-429242 on the SREBP pathway in mock-infected and DENV-2-infected Huh-7.5.1 cells. The amino-pyrrolidine amide compound PF-429242 is a potent and selective catalytic site-directed inhibitor of SKI-1/S1P endoproteolytic activity [13, 25, 34, 39]; and under our experimental conditions, the CC_50_ of PF-429242 in Huh-7.5.1 cells was 81 μM (Fig 1A and [13]).

To evaluate the effects of PF-429242 on the SREBP pathway, Huh-7.5.1 cells were pre-treated with 10 or 20 μM PF-429242 for 24 hours. The inhibitor was removed and the cells were mock-infected or infected with DENV-2 for 48 hours before total RNA was harvested and analyzed by qRT-PCR. We observed a strongly reduced expression of SREBP-1c and SREBP-2 mRNAs in both the mock-infected and DENV-2-infected Huh-7.5.1 cells (Fig 1B and S2 Fig). This result is consistent with previous findings showing that transcription of both SREBP-1c and SREBP-2 is stimulated by SREBPs in a feed-forward mechanism that requires sterol regulatory element (SRE) sequences in the promoters of these genes [40, 41]. As expected, the expression of two of the SREBP target genes, PCSK9 and LDLR, was robustly decreased in both mock-infected and DENV-2-infected Huh-7.5.1 cells treated with PF-429242 compared to control (Fig 1B and S2 Fig). In contrast, expression of two SREBP-independent genes, SKI-1/S1P and furin, was approximately the same in these samples as in controls (Fig 1B and S2 Fig). These results demonstrate that inhibition of SKI-1/S1P enzymatic activity using PF-429242 impairs activation of the SREBP pathway in both mock-infected and DENV-2 infected Huh-7.5.1 cells.

Given that SKI-1/S1P-mediated SREBP proteolytic activation controls the expression of genes directly involved in intracellular fatty acid and cholesterol biosynthesis [28], two important components of LDs, we next tested the hypothesis that pharmacological inhibition of the subtilase SKI-1/S1P by PF-429242 could represent a powerful approach to regulating LD formation in Huh-7.5.1 cells.

To investigate the efficacy of PF-429242 to reduce cytosolic LD formation, we used Nile red, a selective fluorescent stain for intracellular LDs [38]. Untreated, DMSO-treated (vehicle control) and 20 μM PF-429242-treated Huh-7.5.1 stained cells were examined by confocal microscopy (Fig 1C). Analysis of Nile red-stained LDs demonstrated a specific effect of PF-429242 in Huh-7.5.1-treated cells, with an overall 50% reduction in LD-positive areas and LD numbers (Fig 1D). In contrast, LD-positive areas and LD numbers were approximately the same in the DMSO (vehicle control) and untreated cells (Fig 1D). Taken together, these results demonstrate that inhibition of SKI-1/S1P enzymatic activity in Huh-7.5.1 cells using the potent and selective inhibitor PF-429242 impairs activation of the SREBP pathway and results in a decrease in LD formation (LD counts and LD-positive area per cell).

### Huh-7.5.1 cells support DENV-2 replication and DENV capsid protein binding to hepatic lipid droplets

Since the SKI-1/S1P-dependent activation of the SREBP pathway and LD formation can be inhibited with PF-429242, we hypothesized that PF-429242 could act as an antiviral agent against DENV infection in Huh-7.5.1 cells. To test the antiviral properties of PF-429242 in these cells, we first needed to show that Huh-7.5.1 cells support DENV-2 infection. Therefore, Huh-7.5.1 cells were mock-infected or infected with DENV-2 at a MOI of 1. This approach allowed the monitoring of DENV-2 infection in Huh-7.5.1 cells over prolonged periods, whereas at higher MOI a rapid virus-induced cell death is observed thus precluding gene expression profiling of infected cells at the late stage of infection. Total RNA was extracted at 0, 4, 8, 24, 48, and 72 hours post-infection (hpi), and viral replication efficiency was determined by quantifying the expression level of DENV-2 RNA by qRT-PCR. Alternatively, viral infection was demonstrated by visualizing DENV-2 Capsid (C) protein by indirect immunofluorescence.

**Fig 2:**
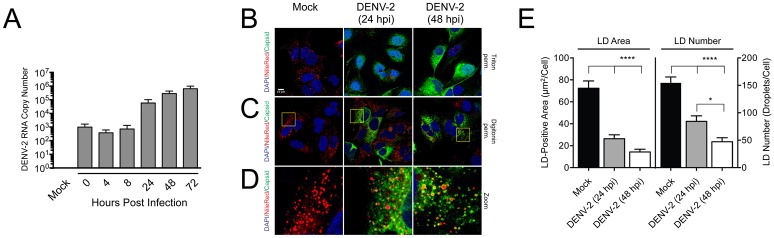
Huh-7.5.1 cells support DENV-2 replication and DENV capsid protein binding to hepatic lipid droplets. (A–E) Huh-7.5.1 cells were infected with DENV-2 (MOI = 1) or mock-infected. (A) Total RNA was extracted at various time-points (0, 4, 8, 24, 48, and 72 h) post-infection, and mRNA expression of DENV-2 RNA was quantified by qRT-PCR. Results were normalized against control β-actin mRNA levels and expressed as fold change relative to the time-matched mock-infected controls. (B–E) Cells were harvested at 24h and 48h post-infection. Cells were fixed with paraformaldehyde and permeabilized with Triton X-100 (B) or digitonin (C). (D) DENV C accumulation around lipid droplets in Huh-7.5.1 cells infected with DENV (MOI = 1) at 48 hpi. Fixed cells were stained for cell nuclei using Hoechst dye (blue), for lipid droplets using Nile red (red), and for DENV using anti-capsid (green). (E) Abundance of LDs was quantified by measuring the average LD-positive area (μm^2^)/cell and the average number of LDs/cell based on Nile red fluorescence in mock-infected cells, DENV-2 infected cells (24 hpi), and DENV-2 infected cells (48 hpi) using Fiji software (>50 cells analyzed). Images were obtained using a Leica SP8 confocal microscope with a 100x objective. Results (mean ± SEM) from at least three independent experiments are shown. Statistical significance was calculated for DENV-2-infected cells compared to mock-infected cells at time-points corresponding to DENV-2-infected cells with a two-way ANOVA with a Bonferroni’s post-test (*, *p* < 0.05; ****, *p* < 0.001).

The temporal expression of DENV-2 RNA is presented in Fig 2A. Newly biosynthesized viral RNA is detected at 24 hpi and continued to increase steadily thereafter reaching the highest level at 72 hpi. Similarly, confocal microscopic analysis of DENV-2 infected cells at 24 and 48 hpi revealed a robust biosynthesis of DENV-2 C protein in most cells that were analyzed using indirect immunofluorescence (Fig 2B and 2C). Under our experimental conditions, when DENV-infected cells were fixed with paraformaldehyde and permeabilized with Triton X-100, the C protein was found in both the nucleus and cytoplasm (Fig 2B). When Huh-7.5.1 cells were permeabilized with digitonin, which, unlike Triton X-100, preferentially permeabilizes the plasma membrane leaving the nuclear envelope intact [42], the C protein was primarily distributed to the cytoplasm (Fig 2C), forming a ring-like shape on the surface of the LDs at 48 hpi (Fig 2D). These results are consistent with previous studies demonstrating that under specific experimental conditions, the subcellular localization of C protein can be detected either in the cytoplasm or in the nucleus of DENV-infected cells [20, 43]. Importantly, reduction of the LD numbers and LD-positive areas in DENV-2-infected cells at 24 and 48 hpi were observed using fluorescence intensity of stained cells with Nile red (Fig 2E). These results are consistent with an earlier study by Heaton *et al*. [18], which demonstrated that DENV-2 infection in Huh-7.5 cells leads to an autophagy-dependent reduction of LDs.

Taken together, our findings demonstrate that Huh-7.5.1 cells support DENV-2 infection, which is consistent with our observation that the DENV capsid protein re-localized to LD in Huh-7.5.1 cells, an important step in the DENV lifecycle [20].

### Pretreatment of Huh-7.5.1 cells with PF-429242 results in a dose-dependent decrease in intracellular DENV-2 NS1 protein abundance and a 3-log decrease in extracellular viral titer

In order to examine the effectiveness of PF-429242 as an anti-DENV agent, Huh-7.5.1 cells were pretreated with increasing concentrations of PF-429242 (0.1 μM to 20 μM) prior to infection with DENV-2. The inhibitor was removed after 24 hours, and the cells were then infected with DENV-2 for 48 hours. Viral protein synthesis in Huh-7.5.1 cells was monitored by determining the level of DENV-2 NS1 protein abundance in total cell lysates using Western blot analysis.

**Fig 3:**
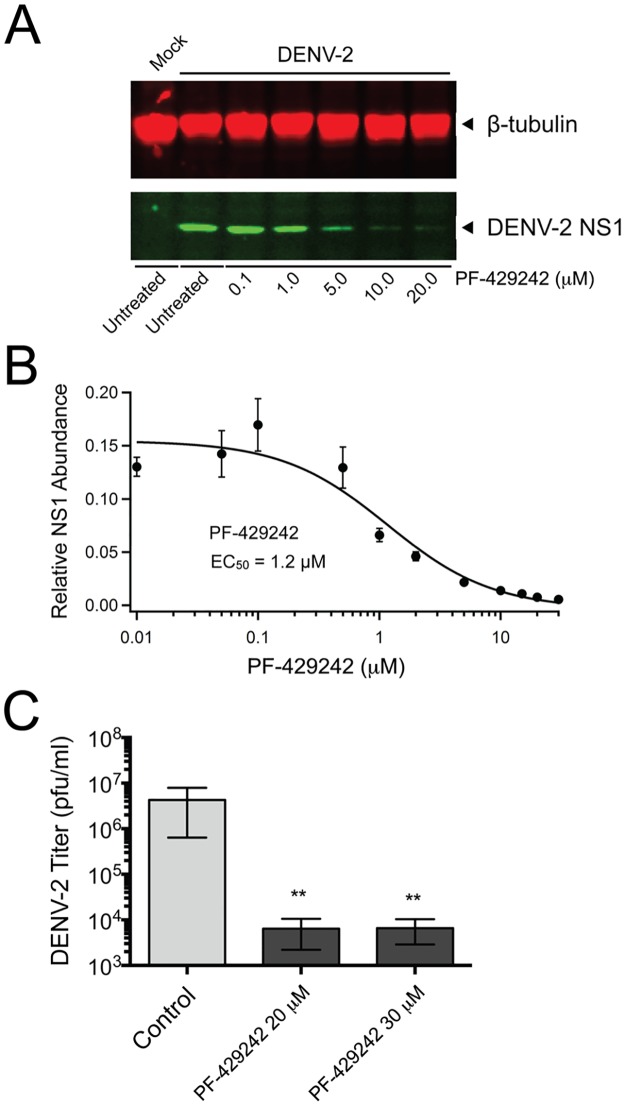
Pretreatment of Huh-7.5.1 cells with PF-429242 results in a dose-dependent decrease in intracellular DENV-2 NS1 protein abundance and a 3-log decrease in extracellular viral titer. (A–B) Huh-7.5.1 cells were either untreated or treated with increasing concentrations of PF-429242 for 24 hours. The inhibitor was removed and the cells were then infected with DENV-2 (MOI = 0.01) for 48 hours. (A) Cell lysates were probed for DENV NS1 (green) and normalized to β-tubulin (red). Representative Western blot for the effect of PF-429242 on DENV NS1 protein level is shown. (B) Dose response curve of normalized, averaged NS1 signal quantified from Western blots of three independent experiments (EC_50_ = 1.2 μM). (C) Huh-7.5.1 cells were treated with increasing concentrations of PF-429242 or with 0.03% DMSO (control) for 24 hours. The inhibitor was removed and the cells were then infected with DENV-2 (MOI = 0.01). 48 hours post-infection, supernatant was collected and viral titer was determined by infecting naïve Vero E6 cells and counting plaques 5 days post-infection. Results (mean ± SEM) from three independent experiments are shown. Statistical significance was calculated for PF-429242-treated cells compared to control with a ratio paired Student’s *t*-test (C) (**, *p* < 0.01).

The host cell pretreatment with PF-429242 resulted in a dose-dependent decrease in the intracellular level of DENV-2 NS1 protein (Fig 3A). A near-complete block of NS1 production was observed following treatment with 20 μM of inhibitor (Fig 3B). Under these experimental conditions, a 19.3-fold reduction in intracellular DENV NS1 was observed when treated with 20 μM PF-429242 (Fig 3B). We found that PF-429242 decreases intracellular DENV-2 NS1 protein abundance in infected cells with an EC_50_ concentration of 1.2 ± 0.2 μM. Furthermore, the Selectivity Index, expressed as the ratio of CC_50_ on EC_50_, indicated that PF-429242 has a high value (>68) and is therefore a good small-molecule antiviral candidate for further studies.

Next, the effect of inhibiting SKI-1/S1P on the formation of infectious DENV virions was investigated by plaque assay. Supernatants from PF-429242 (20 and 30 μM) or DMSO-treated Huh-7.5.1 cells infected with DENV-2 were collected 48 hpi and titrated on naive Vero E6 cells; plaques were counted five days post-infection. Results showed a ~3-log decrease in DENV-2 titer in cells pretreated with 20 or 30 μM PF-429242 compared to the DMSO-treated control (Fig 3C).

These findings clearly demonstrate the antiviral activity of PF-429242 against DENV-2 in Huh-7.5.1 cells: Pretreatment of Huh-7.5.1 cells with PF-429242 results in a dose-dependent inhibition of DENV infection with an EC_50_ of 1.2 μM (CC_50_ of 81 μM and SI of 68) and a ~3-log decrease in DENV-2 titer with 20 μM of PF-429242.

### Pretreatment of Huh-7.5.1 cells with PF-429242 results in a robust decrease in intracellular DENV-2 RNA

**Fig 4:**
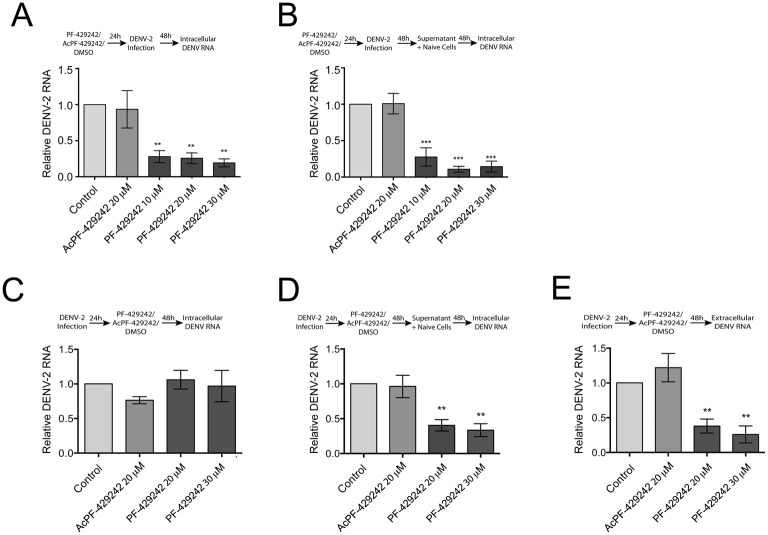
Pretreatment of Huh-7.5.1 cells with PF-429242 results in a robust decrease in intracellular DENV-2 RNA and post-treatment of DENV-2 infected Huh-7.5.1 cells with PF-429242 impairs the assembly and/or release of infectious virus particles. (A–B) Huh-7.5.1 cells were treated either with 20 μM AcPF-429242, with various concentrations of PF-429242 (10–30 μM), or DMSO (0.01–0.03%) (control) for 24 hours. The inhibitor was removed and the cells were then infected with DENV-2 (MOI = 0.01) for 48 hours. (A) Total RNA was harvested and DENV-2 RNA levels, normalized to β-actin transcript levels, were relatively quantified in cell extracts using qRT-PCR. (B) Collected supernatant was cultured with naïve Huh-7.5.1 cells for 48 hours, and DENV-2 RNA levels were quantified. (C–E) Huh-7.5.1 cells were infected with DENV-2 (MOI = 0.01). 24 hours post-infection, cells were treated either with 0.03% DMSO (control), 20 μM AcPF-429242, or 20/30 μM PF-429242 for 48 hours. Total intracellular RNA during primary infection (C) and secondary infection (D), and extracellular RNA during primary infection (E), were harvested and analyzed for DENV RNA levels. Intracellular DENV-2 RNA levels (C, D) were normalized to β-actin transcript levels, while extracellular DENV-2 RNA levels (E) were normalized by volume and then relatively quantified using qRT-PCR. Values represent mean ± SEM of three independent experiments. Statistical significance was calculated compared to control with a one-way ANOVA with a Bonferroni’s post-test (**, *p* < 0.01; ***, *p* < 0.005).

To further dissect the steps in the DENV lifecycle impaired by PF-429242 inhibition of host cell SKI-1/S1P, we examined the relative levels of intracellular DENV-2 RNA in primary and secondary infected cells. First, Huh-7.5.1 cells were pretreated with different concentrations of PF-429242 (10–30 μM), 20 μM AcPF-429242 (inactive analog; S3 Fig), or DMSO (control) for 24 hours before being infected with DENV-2. Under our experimental conditions, neither DMSO nor AcPF-429242 was toxic to Huh-7.5.1 cells (S3 Fig). At 48 hpi, DENV genomic RNA was isolated and relatively quantified in cell extracts using qRT-PCR. DENV-2 RNA levels were normalized to β-actin transcript levels. In agreement with our previous findings on the relative intracellular abundance of DENV NS1 protein (Fig 3) in PF-429242-treated cells, we found that intracellular levels of DENV-2 RNA were also markedly decreased by an average of 74% in the 20 μM PF-429242-treated cells compared to the DMSO-treated and 20 μM AcPF-429242-treated cells (Fig 4A).

Next, to evaluate the effect of PF-429242 on DENV-2 infectious virus particle production and its spread to naïve cells, we performed an assay involving DENV-2 secondary infection (“re-infection assay”). Huh-7.5.1 cells were pretreated with PF-429242 for 24 hours prior to DENV-2 infection. At 48 hpi, supernatants were collected and incubated with naïve Huh-7.5.1 cells for one hour. Following incubation, the supernatants were removed and the cells were supplemented with fresh complete media for 48 hours before DENV-2 RNA was quantified by qRT-PCR. Consistent with our results observed during primary infection of Huh-7.5.1 cells with DENV-2, we measured a statistically significant reduction in DENV-2 RNA during secondary infection following initial PF-429242 treatment, compared to DMSO or AcPF-429242 (Fig 4B).

These results suggest that pretreatment of Huh-7.5.1 cells with PF-429242 may impair viral replication (Fig 4), and it may consequently compromise viral protein biosynthesis (Fig 3A and 3B) and production of infectious DENV-2 virus particles (Fig 3C) in Huh-7.5.1 cells with low cytosolic LD abundance.

### Post-treatment of DENV-2 infected Huh-7.5.1 cells with PF-429242 does not affect intracellular viral RNA abundance, but it does impair the assembly and/or release of infectious virus particles

We next examined whether PF-429242 can impair DENV-2 RNA synthesis when added to Huh-7.5.1 cells with already established infection. To achieve this goal, Huh-7.5.1 cells were first infected with DENV-2 for 24 hours to allow uninterrupted DENV replication and establishment of infection. At the end of the infection period, the Huh-7.5.1 cells were treated with different concentrations of PF-429242 (20/30 μM), AcPF-429242 (20 μM), or DMSO (0.03%) for 48 hours before DENV-2 RNA levels were quantified by qRT-PCR. Interestingly, in contrast to our finding with the pretreatment of Huh-7.5.1 cells with PF-429242 (Fig 4A and 4B), post-treatment of DENV-2- infected Huh-7.5.1 cells with PF-429242 had no effect on the level of intracellular DENV-2 RNA (Fig 4C).

To investigate the potential effects of PF-429242 on the production of infectious DENV-2 virus particles when applied after DENV-2 infection, we examined the intracellular levels of DENV-2 during secondary infection of Huh-7.5.1 naïve cells (Fig 4D). At 48 hours post-treatment with DMSO (Control), AcPF-429242, and PF-429242, supernatants were collected and incubated with naïve Huh-7.5.1 cells for one hour. Following incubation, the supernatants were removed and the cells were supplemented with fresh complete media for 48 hours before intracellular DENV-2 RNA was quantified by qRT-PCR (Fig 4D). We found that DENV-2 RNA was approximately 50% decreased during secondary infection, when naïve Huh-7.5.1 cells were treated with the supernatants from cells that had first been infected for 24 hours, then treated with PF-429242 for 48 hours, compared to supernatants from cells that had first been infected for 24 hours, then treated with AcPF-429242 or DMSO (Fig 4D).

To examine further the potential effects of PF-429242 on the production of extracellular infectious virus particles under these experimental conditions, the supernatants from primary infected cells were analyzed for the presence of extracellular viral RNA. This analysis revealed that extracellular DENV-2 RNA is decreased by more than 50% from cells treated with PF-429242 after established infection compared to cells treated with DMSO and AcPF-429242 (Fig 4E).

While this analysis does not rule out possible effects of PF-429242 on DENV virus assembly, the correlation observed between the impaired secondary infection (Fig 4D) and reduction of extracellular DENV RNA from the primary infection (Fig 4E) suggests that production of extracellular infectious virus particles may be compromised in PF-429242-treated cells. Importantly, pharmacological treatment of already infected DENV-2 cells using PF-429242 resulted in a 50% reduction of DENV extracellular RNA.

Taken together, these results indicate that inhibiting SKI-1/S1P can interrupt the DENV lifecycle at multiple stages of viral infection, both preventing naïve cells from becoming infected and preventing the assembly and/or release of infectious virus particles from already infected cell populations.

### Extracellularly applied oleic acid, an inducer of lipid droplet formation, rescues intracellular DENV-2 RNA abundance in PF-429242-treated Huh-7.5.1 cells

Inhibition of DENV infectivity by PF-429242 suggested that active lipid metabolism in the host cell is important for the viral lifecycle. To determine whether the availability of intracellular fatty acids, and specifically their accumulation in cytosolic LDs, was a limiting factor for DENV-2 infection, Huh-7.5.1 cells treated with PF-429242 were supplemented with an exogenously added fatty acid (oleic acid) to induce LD formation during DENV-2 infection [35].

**Fig 5:**
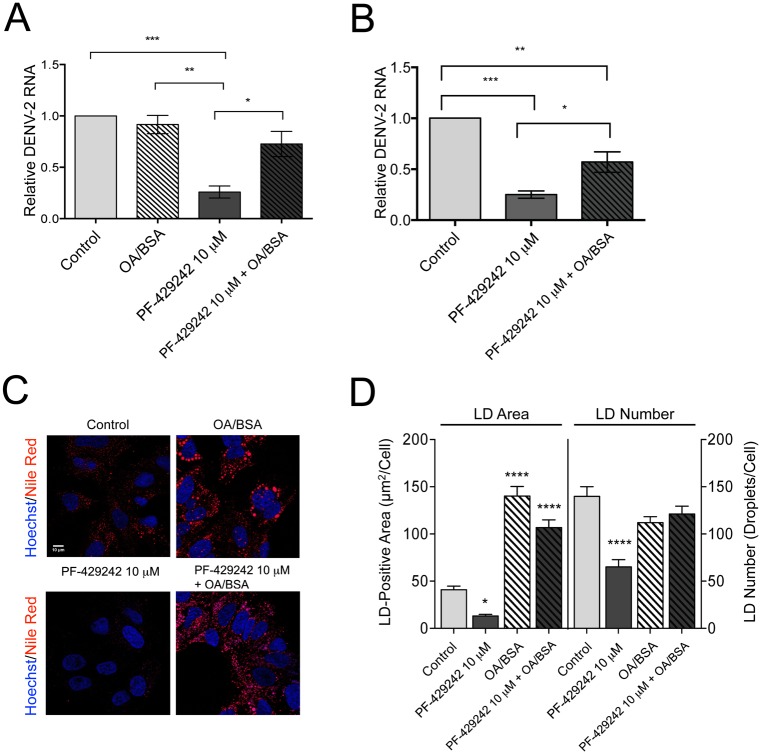
Extracellularly applied oleic acid, an inducer of lipid droplet formation, rescues intracellular DENV-2 RNA abundance in PF-429242-treated Huh-7.5.1 cells. (A–D) Huh-7.5.1 cells were treated either with 0.01% DMSO (control) or 10 μM PF-429242 for 24 hours. The inhibitor was removed and the cells were then infected with DENV-2 (MOI = 0.01) (A–B) or mock-infected (C–D) with or without addition of 0.6 mM oleic acid (with BSA in molar ratio 6:1) for 24 hours. (A) Total RNA was harvested and DENV-2 RNA levels, normalized to β-actin transcript levels, were relatively quantified in cell extracts using qRT-PCR. (B) Collected supernatant was cultured with naïve Huh-7.5.1 cells for 48 hours, and DENV-2 RNA levels were quantified. (C) Representative images of the effect of PF-429242 and oleic acid on lipid droplets are shown. Fixed cells were permeabilized with Triton X-100 and stained for cell nuclei using Hoechst dye and for lipid droplets using Nile red. Images were obtained using a Leica SP8 confocal microscope with a 100x objective. (D) Abundance of LDs was quantified by measuring the average LD-positive area (μm^2^)/cell and the average number of LDs/cell based on Nile red fluorescence in 0.01% DMSO-treated (control), PF-429242-treated (10 μM), OA/BSA-treated, and PF-429242 (10 μM) + OA/BSA-treated cells using Fiji software (>50 cells analyzed). Statistical significance was calculated with a two-way (A, D) and one-way (B) ANOVA with a Bonferroni’s post-test (*, *p* < 0.05; **, *p* < 0.01; ***, *p* < 0.005; ****, *p* < 0.001). Values represent mean ± SEM of three independent experiments.

As we expected, DENV-2 RNA abundance is rescued by the addition of oleic acid/BSA in cells treated with 10 μM PF-429242 compared to cells treated with PF-429242 alone (Fig 5A) at 24 hpi. To further investigate the potential rescue effect of oleic acid/BSA on infectious DENV-2 particles release and DENV spread to naïve cells, we examined the intracellular levels of DENV-2 RNA during secondary infection of Huh-7.5.1 naïve cells (Fig 5B). We found that DENV-2 RNA was more than 2.2-fold higher during secondary infection in cells treated with the supernatants from primary infection (Fig 5A) that were supplemented with oleic acid/BSA and 10 μM PF-429242 compared to 10 μM PF-429242 alone. The incomplete rescue of DENV-2 RNA abundance observed with oleic acid/BSA during the re-infection assay may be due to viral reliance on other SKI-1/S1P-dependent cellular functions besides LD formation [44, 45].

Interestingly, using the relative fluorescence intensity of Nile red (Fig 5C), we observed that oleic acid is a regulator of LD-positive areas but not LD number in Huh-7.5.1 cells (Fig 5D), which means that treatment of Huh-7.5.1 cells with oleic acid only increased LD area under our experimental conditions. Since there was no significant increase in DENV RNA levels detected in virally infected cells in oleic acid/BSA-treated cells compared to control-treated cells (Fig 5A), these results suggest that the main molecular determinant of DENV-2 viral RNA abundance in Huh-7.5.1 cells is the number of LDs, not the LD area per cell.

Overall, these results obtained with oleic acid treatment demonstrate that the inhibitory effect of PF-429242 on DENV-2 infection is attributed to its intrinsic capacity to robustly reduce total intracellular lipid levels, specifically triglycerides and cholesterol esters [13], two major constituents of cellular LDs [46]. These findings were confirmed by using Nile red (Fig 5C and 5D), a dye selective for neutral lipids such as triglycerides and cholesterol esters that make up the core of an LD [24, 38]. Importantly, our results reveal human SKI-1/S1P as a regulator of LD formation and further confirm that LDs are necessary for DENV-2 infection of human hepatoma Huh-7.5.1 cells.

### Pretreatment of Huh-7.5.1 cells with PF-429242 results in a robust decrease in intracellular viral RNA for all four DENV serotypes

**Fig 6:**
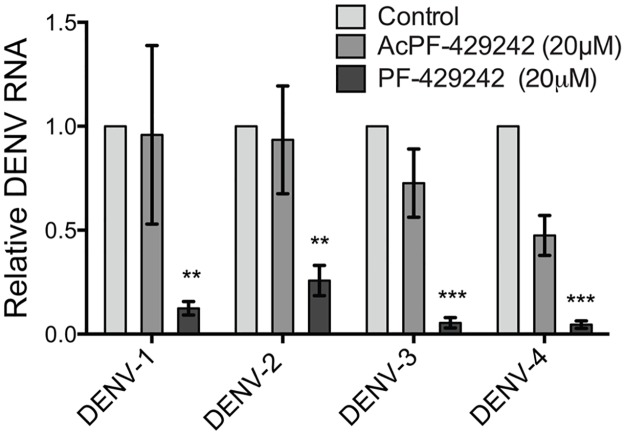
Pretreatment of Huh-7.5.1 cells with PF-429242 results in a robust decrease in intracellular viral RNA for all four DENV serotypes. Huh-7.5.1 cells were treated either with 0.02% DMSO (control), 20 μM AcPF-429242, or 20 μM PF-429242 for 24 hours. The inhibitor was removed and the cells were then infected with one of the four DENV serotypes (MOI = 0.01) for 48 hours. Total RNA was harvested and DENV RNA levels of four serotypes, normalized to β-actin transcript levels, were relatively quantified in cell extracts using qRT-PCR. Values represent mean ± SEM of three independent experiments. Statistical analysis was performed for PF-429242 and AcPF-429242 treated cells compared to control with a two-way ANOVA with a Bonferroni’s post-test (**, *p* < 0.01; ***, *p* < 0.005).

Finally, to establish whether replication with other DENV serotypes can be inhibited by PF-429242 in the same manner as DENV-2, Huh-7.5.1 cells were pretreated with 20 μM of PF-429242, DMSO (control) or AcPF-429242 for 24 hours. The inhibitor was removed and the cells were then infected with DENV-1, -2, -3, or -4 (MOI = 0.01) for 48 hours. Total cellular RNA was harvested, and DENV-1, -2, -3, and -4 RNA levels, normalized to β-actin transcript levels, were relatively quantified in cell extracts using qRT- PCR. Results showed that DENV-1 RNA was reduced by 84%, DENV-2 by 74%, DENV-3 by 95%, and DENV-4 by 95% compared to control-treated cells (Fig 6). These results demonstrate that inhibiting the enzymatic activity of human subtilase SKI-1/S1P using PF-429242 dramatically reduces intracellular viral RNA abundance of all four DENV serotypes in Huh-7.5.1 cells.

## Discussion

In this study, using PF-429242, an active-site-directed inhibitor of SKI-1/S1P, we demonstrated that strategic manipulation of human SKI-1/S1P enzymatic activity in Huh-7.5.1 human hepatoma cells provides a means of interfering with the SKI-1/S1P-mediated proteolytic activation of the SREBP pathway. PF-429242 treatment in Huh-7.5.1 cells results in a robust inhibition of cytosolic LD formation and effectively inhibits viral infection of Huh-7.5.1 cells by all four DENV serotypes (1–4). Collectively, our results demonstrate that human SKI-1/S1P is a potential target for indirect-acting pan-serotypic anti-dengue virus agents, and these results reveal new therapeutic opportunities associated with the use of lipid-modulating drugs for controlling DENV infection.

### Human subtilase SKI-1/S1P is a regulator of lipid droplet formation in Huh-7.5.1 human hepatoma cells

As hypothesized, inhibiting the SKI-1/S1P dependent activation of the SREBP pathway and expression of SREBP-activated genes resulted in a robust decrease in cytoplasmic LD formation in PF-429242-treated cells. Our results demonstrated that pharmacological inhibition of the subtilase SKI-1/S1P by PF-429242 represents a powerful approach to regulating the LD number and area in Huh-7.5.1 cells. Interestingly, identification of human SKI-1/S1P as a regulator of LD formation is consistent with the identification of the SREBP and SCAP as key genes involved in LD formation using a genome-wide RNA interference screen in *Drosophila* S2 cells [47]. Collectively, these observations suggest potential therapeutic opportunities associated with the use of SKI-1/S1P inhibitors to develop a new class of lipid droplet-modulating agents.

### Strategic manipulation of human SKI-1/S1P enzymatic activity provides a means of effectively inhibiting viral infection of Huh-7.5.1 cells by DENV-2

Lipids and cholesterol have previously been reported as playing important roles in the DENV lifecycle [17, 18, 20, 21, 48]. The viral NS3 serine protease was implicated in recruiting fatty acid synthase to the replicase complex, leading to increased cellular fatty acid synthesis [48]. Additionally, LDs were found to be necessary during the viral assembly steps, as the capsid protein of DENV is recruited to LDs and this step is crucial for forming infectious viral particles [20]. Furthermore, degradation of LDs by autophagy for ATP generation is necessary to support viral replication [18]. Cholesterol in the viral envelope was proposed as being important in the fusion process for DENV entry, while intracellular cholesterol transport is necessary both for viral entry and replication [17, 21]. However, although the collective significance of lipids and cholesterol in most steps of the DENV lifecycle has been recognized, it is still not clear whether the targeting of major metabolic lipid and cholesterol pathways, such as the SREBP pathway, is a viable option for developing host-directed therapeutics for DENV infection.

In this study, we demonstrated that inhibition of the SKI-1/S1P-dependent activation of the SREBP pathway and SREBP-activated genes using PF-429242 resulted in a dose-dependent decrease in DENV-2 infection in Huh-7.5.1 cells (Fig 3). Importantly, the antiviral effect of PF-429242 is associated with a decrease in cytosolic LD numbers and LD-positive areas (Fig 1). The anti-DENV activity of PF-429242 is very robust and characterized by strong reductions in DENV NS1 protein abundance, reduction in intracellular and extracellular DENV RNA, and formation of infectious virions (Figs 3 and 4). Of note, we reported previously that PF-429242 treatment of HCV-infected Huh-7.5.1 cells inhibited HCV infection with an EC_50_ of 6.4 μM [13]. Here, the same treatment led to a reduction in DENV-2 infection with an EC_50_ of 1.2 μM, showing that inhibition of SKI-1/S1P has a more potent antiviral effect against DENV-2 than HCV.

Using a lipid complementation assay, we demonstrated that the antiviral effects of PF-429242 can be reduced by adding exogenous oleic acid to DENV-infected cells (Fig 5A and 5B). We demonstrated that the total LD-positive area per cell is increased in response to oleic acid in untreated and PF-429242-treated Huh-7.5.1 cells (Fig 5C and 5D). These findings are consistent with the prior work of Rohwedder et al. using lipidomic analysis of oleic acid-treated Huh-7 cells that showed an increase in triglyceride and cholesterol ester content in response to oleate stimulation [49]. In addition, we previously reported that PF-429242 treatment of Huh-7.5.1 cells reduced cholesterol ester levels by 63% and total intracellular triglycerides by 51%, two major constituents of cellular LDs [13]. Collectively, these observations indicate that the anti-dengue activity of PF-429242 is associated with the inhibition of SKI-1/S1P-mediated activation of the SREBP transcriptional network, a novel important host pathway for LD formation and the DENV lifecycle.

In addition, since the SREBP pathway regulates cholesterol biosynthesis and cholesterol plays a significant role in the DENV lifecycle [16, 17, 31], inhibition of SKI-1/S1P by PF-429242 could interfere with viral infection by depleting intracellular cholesterol levels. This is best exemplified by the recent reports of Petersen et al. [50] and Kleinfelter et al. [51] that demonstrated the critical roles of the SREBP cholesterol regulatory pathway for the viral lifecycle of hantaviruses using the SKI-1/S1P inhibitor, PF-429242.

Finally, our observation that post-treatment of DENV-2-infected Huh-7.5.1 cells with PF-429242 does not affect intracellular viral RNA synthesis indicated that DENV replication was not compromised; however, extracellular viral RNA and viral RNA levels during secondary infection were still reduced (Fig 4E and 4D, respectively), suggesting that the production and/or release of infectious virus particles had been compromised in the virally infected cells. This also implies that existing intracellular fatty acid and cholesterol levels were sufficient to support the establishment of the primary infection but that the post-replication steps (e.g., virion assembly, maturation, release, or re-entry into naïve cells) were compromised. Since reduction in extracellular viral RNA and intracellular viral RNA during secondary infection was comparable relative to control treated cells, re-entry into naïve cells was not the factor that was compromised by PF-429242 treatment. These findings are in agreement with a study by Samsa et al. reporting that DENV assembles capsids on LDs [20]. Accordingly, SKI-1/S1P inhibition by PF-429242 in Huh-7.5.1 cells, which leads to reduction in LD number and area would impair assembly and release of infectious virus particles.

Our results are in partial agreement with those of Uchida et al., who recently reported suppressive effects of PF-429242 on DENV propagation in HeLa cells derived from cervical cancer cells [52]. Whereas the first part of the work by Uchida and collaborators agrees with our findings on the pan-serotypic inhibitory effect of PF-429242 on DENV infection (Fig 6), we reached an opposing conclusion on the mechanism of action of PF-429242. Uchida et al. concluded that the PF-429242-associated depletion of LD and cholesterol in HeLa cells are not direct causes of the virus inhibition. In our results, however, SKI-1/S1P inhibition by PF-429242 in human hepatoma Huh-7.5.1 cells was reversed by exogenous oleic acid, which acts as an inducer of LD formation in PF-429242-treated cells. These discrepancies may be related to differences in the cell line studied. We selected human hepatoma Huh-7.5.1 cells as the model system to examine the molecular functions of SKI-1/S1P, a key regulator of the lipid homeostasis/SREBP pathway, in the formation of cellular lipid storage droplets and the DENV lifecycle. Cell-intrinsic differences in LD biology between cervical cancer cells and human hepatoma cells could account for the differing results [35]. In addition, the concentration of supplemented oleic acid, the addition of BSA during the oleic acid treatment, and the time of addition of oleic acid during the rescue experiment are other key differences between our study and Uchida et al. In this regard, a study by Ricchi et al. demonstrated concentration- and time-dependent effects of extracellularly applied oleic acid added to hepatic cell cultures on the accumulation of triglycerides and SREBP-1 activation [53]).

### Inhibition of the SKI-1/S1P-mediated proteolytic activation of the SREBP pathway has a pan-serotypic inhibitory effect on DENV infection

Here, we show that infection of all DENV serotypes depends on human SKI-1/S1P enzymatic activity and that its inhibition has a pan-serotypic inhibitory effect on the DENV lifecycle (Fig 6). Infection with any of the DENV serotypes may develop into non-severe dengue fever (DF) or into life-threatening dengue haemorrhagic fever (DHF) and dengue shock syndrome (DSS). It has been observed that DHF and DSS more often result from secondary infection with a heterologous DENV serotype [54]. After infection with one serotype, an individual develops immunity to all four serotypes but only for a short period of time. The immunity to other serotypes usually wanes over two to three months, leaving life-long immunity established only against the primary infecting serotype [55]. Subsequent exposure to heterologous serotypes increases an individual’s risk of developing DHF/DSS, which is believed to arise from a phenomenon known as *antibody-dependent enhancement* (ADE) [56, 57]. Since all four DENV serotypes are now circulating in most tropical and subtropical areas of the world and are endemic in over 100 countries, candidates for DENV therapeutic agents need to be able to target all DENV serotypes [3].

### SKI-1/S1P is a potential target for indirect-acting antiviral agents against DENV infection

One of the advantages of targeting a cellular enzyme for antiviral therapy is that it dramatically reduces the likelihood of nascent antiviral resistance. Here, PF-429242 targets all DENV serotypes with similar efficacy and may be useful as a broad-spectrum antiviral against other viruses that depend on lipid homeostasis/SREBP pathways for their lifecycles. Although cellular SKI-1/S1P activity plays an important role in preventing aberration of lysosomal functions [58], the ER stress response [59], and bone mineralization [60, 61], its short-term inhibition in the case of acute infections by DENV and other LD-dependent microbes may represent a viable therapeutic strategy in DENV-associated disease [62].

## Supporting information

S1 Fig Oligonucleotide primers and fluorogenic probes used in the serotype-specific DENV virus real-time RT-PCR assay. Probes were hybridized with the HEX fluorophore, and the black hole quencher-1 (BHQ-1) was used as the fluorophore-quencher.
(PDF)

S2 Fig Inhibition of SKI-1/S1P using PF-429242 prevents activation of the SREBP pathway in DENV-2 infected Huh-7.5.1 cells. Huh-7.5.1 cells were treated either with 0.02% DMSO (control) or 10/20 μM PF-429242 for 24 hours. The inhibitor was removed and the cells were then infected with DENV-2 (MOI = 0.01) for 48 hours. Total RNA was extracted and the mRNA levels of SREBP-1c, SREBP-2, PCSK9, LDLR, FURIN, and SKI-1/S1P were quantified by qRT-PCR in DENV-2-infected cells. Statistical significance was calculated with a two-way ANOVA with Bonferroni’s post-test. Results were normalized against β-actin mRNA levels and expressed as fold change. Values represent average ± SEM of three independent experiments (**, *p* < 0.01; ****, *p* < 0.001).
(PDF)

S3 Fig AcPF-429242 is not cytotoxic in Huh-7.5.1 cells. Chemical structure of PF-429242 acetyl derivative (AcPF-429242) is shown. AcPF-429242 was evaluated for cytotoxicity in Huh-7.5.1 cells. Huh-7.5.1 cells were treated with DMSO (0.01% and 0.02%) or AcPF-429242 (10 μM and 20 μM) for 24 hours before the inhibitor was removed and fresh complete media was added to the cells for an additional 48 hours. The relative cytotoxicity of the compounds was then determined using an MTS-based cell viability assay. The absorbance measured at 490 nm is proportional to the number of living cultured cells. Results (mean ± SEM) from three independent experiments are shown. Statistical significance was calculated with a one-way ANOVA with Bonferroni’s post-test.
(PDF)
