# Supplementary material for: Human Subtilisin Kexin Isozyme-1 (SKI-1)/Site-1 Protease (S1P) regulates cytoplasmic lipid droplet abundance: A potential target for indirect-acting anti-dengue virus agents
Source: PLoS One. 2017 Mar 24;12(3):e0174483. doi: 10.1371/journal.pone.0174483 (PMC5365115; doi:10.1371/journal.pone.0174483)
Supplement: S1 Fig — Probes were hybridized with the HEX fluorophore, and the black hole quencher-1 (BHQ-1) was used as the fluorophore-quencher. (PDF) [file pone.0174483.s001.pdf]

S1 Fig. Oligonucleotide primers and fluorogenic probes used in the serotype-specific DENV virus real-time RT-PCR assay

Virus serotype detected	Nucleotide sequence	Genome position	Fluorophore-quencher combination
**DENV-1 F**	CAAAAGGAAGTCGTGCAATA	8973	HEX/BHQ-1
**DENV-1 C**	CTGAGTGAATTCTCTCTACTGAACC	9084	
**DENV-1 probe**	CATGTGGTTGGGAGCACGC	8998	
**DENV-2 F**	CAGGTTATGGCACTGTCACGAT	1605	HEX/BHQ-1
**DENV-2 C**	CCATCTGCAGCAACACCATCTC	1583	
**DENV-2 probe**	CTCTCCGAGAACAGGCCTCGACTTCAA	1008	
**DENV-3 F**	GGACTGGACACACGCACTCA	740	HEX/BHQ-1
**DENV-3 C**	CATGTCTCTACCTTCTCGACTTGTCT	813	
**DENV-3 probe**	ACCTGGATGGATGTCGGCTGAAGGAGCTTG	762	
**DENV-4 F**	TTGTCCTAATGATGCTGGTCG	904	HEX/BHQ-1
**DENV-4 C**	TCCACCTGAGACTCCTTCCA	992	
**DENV-4 probe**	TTCCTACTCCTACGCATCGCATTCCG	960	
