# Supplementary material for: Human Subtilisin Kexin Isozyme-1 (SKI-1)/Site-1 Protease (S1P) regulates cytoplasmic lipid droplet abundance: A potential target for indirect-acting anti-dengue virus agents
Source: PLoS One. 2017 Mar 24;12(3):e0174483. doi: 10.1371/journal.pone.0174483 (PMC5365115; doi:10.1371/journal.pone.0174483)

S2 Fig. Inhibition of SKI-1/S1P using PF-429242 prevents activation of the SREBP pathway in DENV-2 infected Huh-7.5.1 cells.

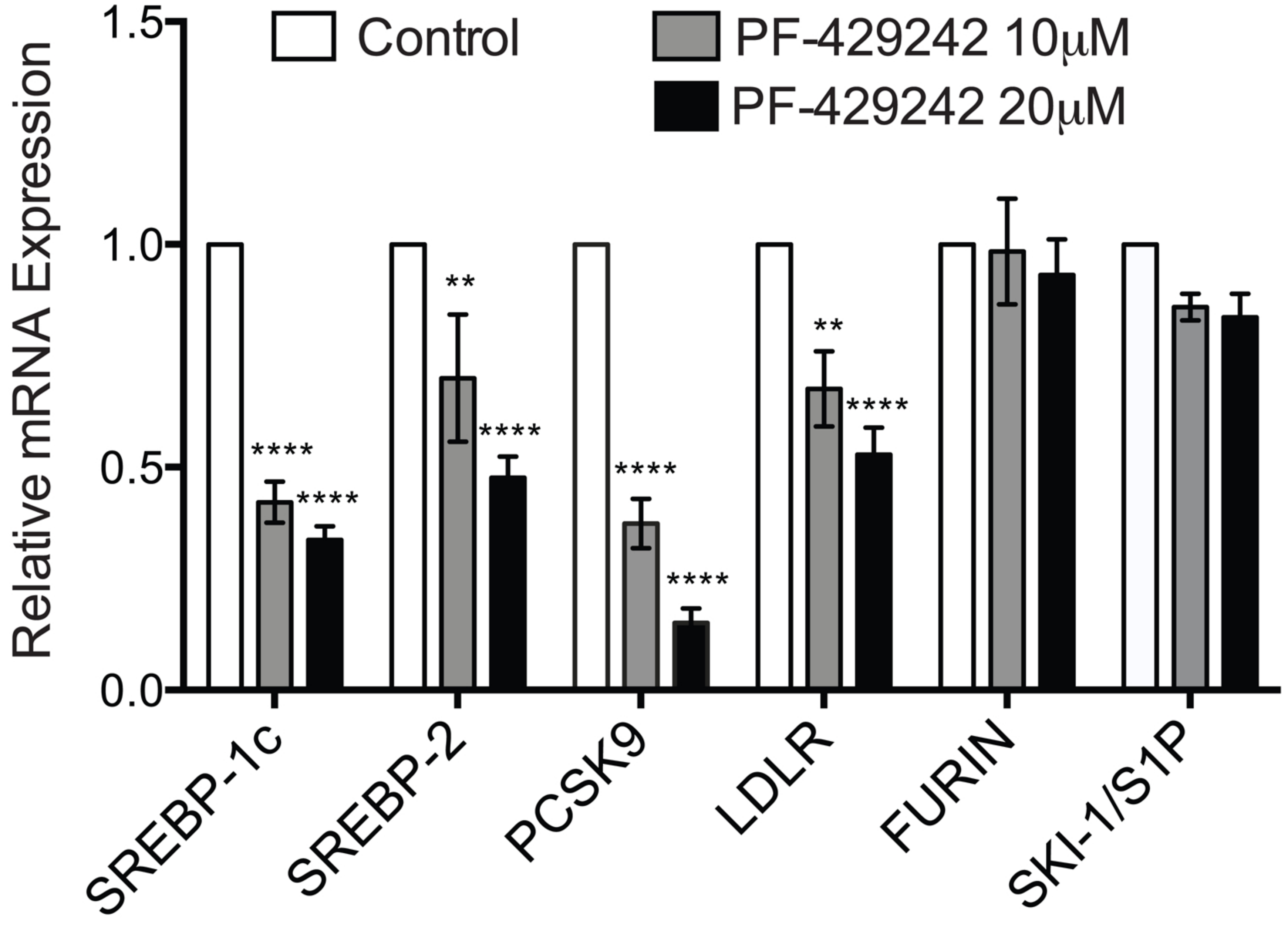

Supplement: S2 Fig — Huh-7.5.1 cells were treated either with 0.02% DMSO (control) or 10/20 μM PF-429242 for 24 hours. The inhibitor was removed and the cells were then infected with DENV-2 (MOI = 0.01) for 48 hours. Total RNA was extracted and the mRNA levels of SREBP-1c, SREBP-2, PCSK9, LDLR, FURIN, and SKI-1/S1P were quantified by qRT-PCR in DENV-2-infected cells. Statistical significance was calculated with a two-way ANOVA with Bonferroni’s post-test. Results were normalized against β-actin mRNA levels and expressed as fold change. Values represent average ± SEM of three independent experiments (**, p < 0.01; ****, p < 0.001). (PDF) [file pone.0174483.s002.pdf]
