# Supplementary figures and images for: Human Subtilisin Kexin Isozyme-1 (SKI-1)/Site-1 Protease (S1P) regulates cytoplasmic lipid droplet abundance: A potential target for indirect-acting anti-dengue virus agents
Source: PLoS One. 2017 Mar 24;12(3):e0174483. doi: 10.1371/journal.pone.0174483 (PMC5365115; doi:10.1371/journal.pone.0174483)

**S3 Fig. AcPF-429242 is not cytotoxic in Huh-7.5.1 cells.**

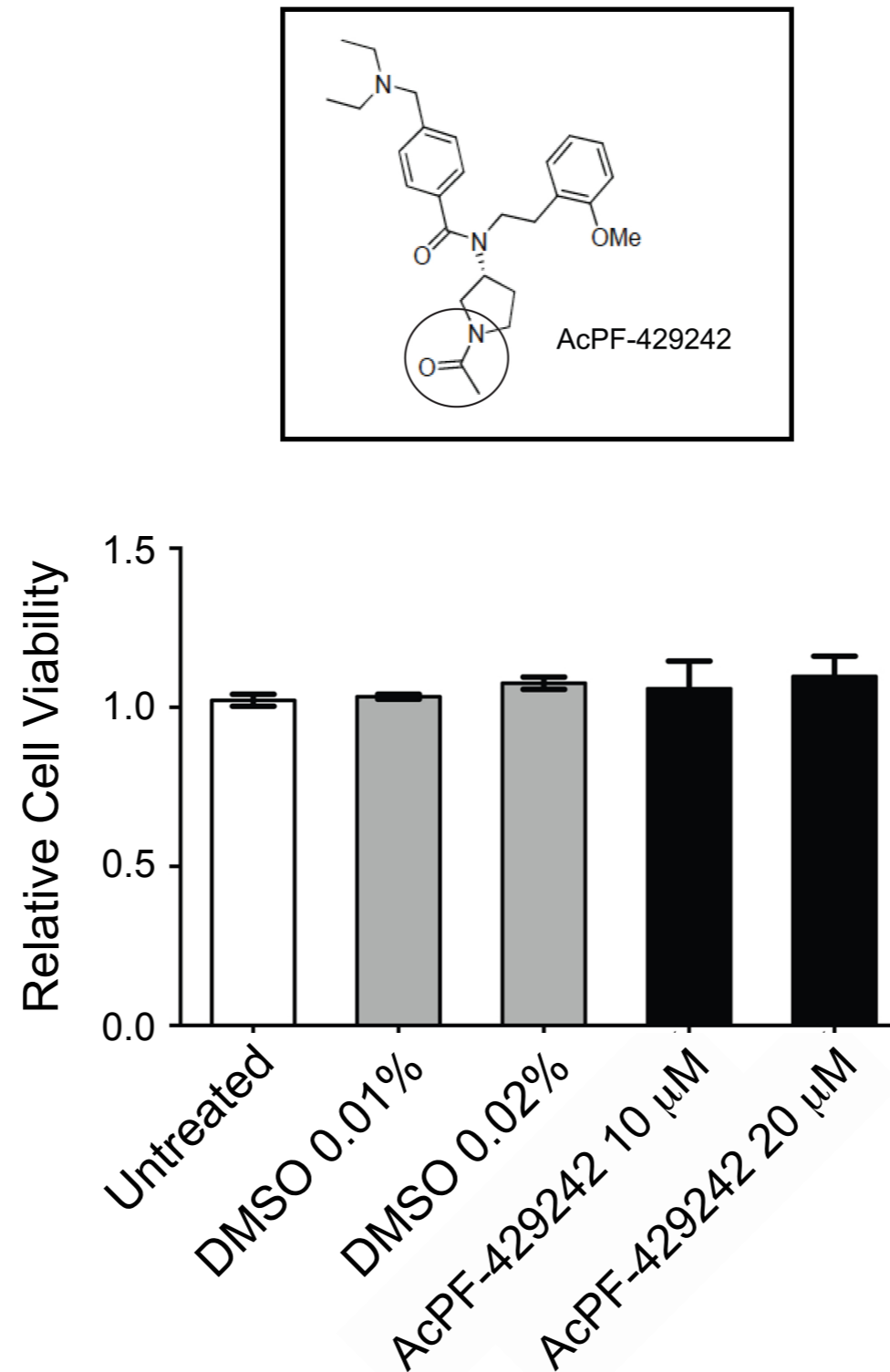

Supplement: S3 Fig — Chemical structure of PF-429242 acetyl derivative (AcPF-429242) is shown. AcPF-429242 was evaluated for cytotoxicity in Huh-7.5.1 cells. Huh-7.5.1 cells were treated with DMSO (0.01% and 0.02%) or AcPF-429242 (10 μM and 20 μM) for 24 hours before the inhibitor was removed and fresh complete media was added to the cells for an additional 48 hours. The relative cytotoxicity of the compounds was then determined using an MTS-based cell viability assay. The absorbance measured at 490 nm is proportional to the number of living cultured cells. Results (mean ± SEM) from three independent experiments are shown. Statistical significance was calculated with a one-way ANOVA with Bonferroni’s post-test. (PDF) [file pone.0174483.s003.pdf]
